# The Role of Obstructive Sleep Apnea and CPAP Therapy in the Functional Hypogonadism of Male Patients With Severe Obesity

**DOI:** 10.1210/clinem/dgaf635

**Published:** 2025-11-24

**Authors:** Alessandro Amodeo, Biagio Cangiano, Nicoletta Del Duca, Alessandro Chilà, Antonio Musolino, Ilinka Garavaglia, Elisa Delle Donne, Alice Casiraghi, Chiara Manzini, Valeria Vezzoli, Daniele Sola, Ilaria Gentile, Alfonso Palo, Giovanni Goggi, Silvia Federici, Luca Persani, Massimo Scacchi, Marco Bonomi

**Affiliations:** Department of Medical Biotechnology and Translational Medicine, University of Milan, Milan 20090, Italy; Department of Medical Biotechnology and Translational Medicine, University of Milan, Milan 20090, Italy; Division of General Medicine, San Giuseppe Hospital, Istituto Auxologico Italiano–IRCCS, Verbania 28824, Italy; Division of General Medicine, San Giuseppe Hospital, Istituto Auxologico Italiano–IRCCS, Verbania 28824, Italy; Department of Medical Biotechnology and Translational Medicine, University of Milan, Milan 20090, Italy; Department of Medical Biotechnology and Translational Medicine, University of Milan, Milan 20090, Italy; Department of Medical Biotechnology and Translational Medicine, University of Milan, Milan 20090, Italy; Department of Medical Biotechnology and Translational Medicine, University of Milan, Milan 20090, Italy; Department of Medical Biotechnology and Translational Medicine, University of Milan, Milan 20090, Italy; Department of Medical Biotechnology and Translational Medicine, University of Milan, Milan 20090, Italy; Division of Endocrine and Metabolic Diseases, Istituto Auxologico Italiano–IRCCS, Milan 20149, Italy; Laboratory of Metabolic Research, San Giuseppe Hospital, Istituto Auxologico Italiano, IRCCS, Verbania 28824, Italy; Department of Translational Medicine, Università del Piemonte Orientale, Novara 28100, Italy; Department of Medical Biotechnology and Translational Medicine, University of Milan, Milan 20090, Italy; Division of Endocrine and Metabolic Diseases, Istituto Auxologico Italiano–IRCCS, Milan 20149, Italy; Biometra, University of Milan, Milan 20122, Italy; Division of Endocrine and Metabolic Diseases, Istituto Auxologico Italiano–IRCCS, Milan 20149, Italy; Department of Medical Biotechnology and Translational Medicine, University of Milan, Milan 20090, Italy; Division of Endocrine and Metabolic Diseases, Istituto Auxologico Italiano–IRCCS, Milan 20149, Italy; Department of Medical Biotechnology and Translational Medicine, University of Milan, Milan 20090, Italy; Division of Endocrine and Metabolic Diseases, Istituto Auxologico Italiano–IRCCS, Milan 20149, Italy; Division of General Medicine, San Giuseppe Hospital, Istituto Auxologico Italiano–IRCCS, Verbania 28824, Italy; Department of Clinical Sciences and Community Health, University of Milan, Milan 20122, Italy; Department of Medical Biotechnology and Translational Medicine, University of Milan, Milan 20090, Italy; Division of Endocrine and Metabolic Diseases, Istituto Auxologico Italiano–IRCCS, Milan 20149, Italy

**Keywords:** obstructive sleep apnea syndrome, functional hypogonadism, obesity, continuous positive airway pressure (CPAP)

## Abstract

**Context:**

Only a few studies have reported a correlation between severe obstructive sleep apnea syndrome (OSAS) and hypogonadism in patients with obesity, regardless of body mass index (BMI). However, longitudinal studies exploring the role of continuous positive airway pressure (CPAP) on gonadal function are scanty.

**Objective:**

This work aimed to investigate in men with severe/complicated obesity the role of OSAS in decreasing testosterone levels and evaluate the effects of CPAP on hormonal status.

**Methods:**

This cross-sectional study consecutively enrolled 204 male inpatients with complicated severe obesity, without known hypogonadism. Polysomnography (or overnight oximetry during CPAP) and blood tests for inflammation and metabolic and hormonal profiles were performed. “Decompensated OSAS” was defined as an Apnea/Hypopnea Index (AHI) in newly diagnosed, or Oxygen Desaturation Index (ODI) in treated patients, above 30 events/hour. A multiple linear regression was implemented to identify the independent factors correlated with total testosterone (TT). Lastly, a longitudinal study of 14 newly diagnosed patients was performed to evaluate the effects of CPAP on TT after 3 months of treatment.

**Results:**

A total of 127 of 204 patients showed low TT (≤10.4 nmol/L). BMI, type 2 diabetes, C-reactive protein, and decompensated OSAS were independently associated with TT (*P* = .039; *P* = .006, *P* = .003, and *P* = .014, respectively). After 3 months of CPAP therapy, TT was higher (*P* = .009) and ODI was associated with such improvement, independently of BMI (*P* = .04).

**Conclusion:**

Decompensated OSAS was found to correlate with low testosterone in men with severe obesity. Moreover, CPAP therapy was shown to improve TT independently of BMI changes.

In 2022, nearly 1 billion adults were living with obesity worldwide. As reported by the World Health Organization, obesity is a chronic, complex disease defined by excessive fat deposits that can impair health (1). Indeed, this condition is associated with higher mortality and morbidity rates, particularly from cardiovascular disease, diabetes, and cancer (2). Regarding hormonal alterations, obesity may be both a cause and a consequence of endocrine disorders, and among them low testosterone levels have been well characterized (3, 4). Male hypogonadism has been reported in up to 45% of patients with moderate-severe obesity when considering total testosterone (TT) and/or free testosterone (FT) concentrations (5). Several mechanisms have been proposed to explain this correlation: While an increase in aromatase activity in adipose tissue and rise in estradiol levels have not been confirmed in recent studies, insulin and leptin resistance and proinflammatory adipocytokines may suppress the hypothalamic-pituitary-gonadal (HPG) axis, at least in part, via effects on kisspeptin neuron modulation (6). Finally, obesity has been associated with obstructive sleep apnea syndrome (OSAS), a condition characterized by recurrent episodes of partial or complete upper airway collapse during sleep, resulting in reduced (hypopnea) or absent (apnea) airflow, and accompanied by cortical arousal, decrease in blood oxygen saturation, loud snoring, and daytime sleepiness (7).

To date, whether OSAS also contributes to the decrease in T blood values in male patients with obesity has remained unclear.

Already in 1989, in a cross-sectional study of men undergoing sleep evaluations, Grunstein et al (8) had found that FT, TT, and sex hormone–binding globulin (SHBG) levels were significantly lower in relation to the severity of sleep apnea; this decrease was independent of the effects of aging and adiposity by covariance analysis. In a longitudinal study, the same authors showed that plasma TT and SHBG, but not FT, significantly increased after 3 months of nasal continuous positive airway pressure (CPAP) treatment (8).

In subsequent studies conducted in small cohorts of patients with obesity, a statistically significant inverse correlation between OSAS and T levels, independent of body mass index (BMI), was confirmed (9, 10), and the severity of hypoxia during sleeping hours was suggested as an additional factor in reducing T levels (11). A recent work confirmed the association between severity of OSAS and sex hormonal profile in a larger cohort of male patients with obesity, revealing that both Apnea Hypopnea Index (AHI) and Oxygen Desaturation Index (ODI) were in significant inverse correlation with TT and FT concentrations after adjustment for age and BMI (12).

The mechanisms by which OSAS would lead to reduced T levels are far from being elucidated, but hypoxia and changes in sleep architecture have been reported to impair the gonadal axis and gonadotropin secretion (13, 14).

The success of CPAP therapy in restoring or improving gonadal function has been investigated, but results are inconclusive. A recent meta-analysis including 12 studies did not confirm the positive effect of CPAP therapy on serum T and gonadotropin levels in male patients with OSAS syndrome, suggesting strategies other than CPAP to treat hypogonadism in such patients (15). Nevertheless, to the best of our knowledge, the effects of CPAP therapy on hormonal outcome have never been evaluated on a population of only patients with obesity.

Therefore, the aim of the present study was to investigate the relationship between obesity, OSAS syndrome, and hypogonadism in the largest possible case series of patients with severe/complicated obesity and without ongoing T replacement therapy, by evaluating the factors associated with hypogonadism. In particular, we investigated (1) the role of the severity/decompensation of OSAS syndrome in the decrease in blood T concentration, adjusting for the other known factors associated with functional hypogonadism in obesity and, (2) the effects of a 3-months-successful CPAP therapy on the gonadal function of a subset of newly diagnosed patients with OSAS.

## Materials and Methods

### Study Design

This monocentric, cross-sectional study involved 204 male patients with obesity admitted between January 2022 and October 2023 to the San Giuseppe Hospital Centre in Piancavallo (Piemonte, Italy), of the Istituto Auxologico Italiano. Male patients with grade II complicated obesity (ie, BMI ≥ 35 and at least 1 of the following: OSAS, cardiovascular disease, decompensated type 2 diabetes mellitus (T2DM), eating disorder, and severe osteoarticular complications) or grade III obesity (ie, BMI ≥ 40) were eligible for inclusion. Patients with already known organic disease affecting the hypothalamic/pituitary region, or systemic disease, or undergoing treatment with glucocorticoids or other drugs interfering with the gonadal axis, were excluded.

In a subset of 14 patients with a new diagnosis of severe OSAS and good compliance with ventilation therapy, we also carried out a longitudinal evaluation of the effects of CPAP on T values after 3 months of treatment.

The study was approved by the institutional review board (Istituto Auxologico Italiano, 05C308) and all patients provided written informed consent to allow their deidentified or anonymized data to be used for research purposes.

### Procedures

At the time of admission, the following anamnestic data were collected: presence of a previous diagnosis of OSAS and concomitant CPAP or bi-PAP therapy; presence or absence of a previous psychiatric diagnosis of binge-eating disorder (BED), according to Diagnostic and Statistical Manual of Mental Disorders (Fifth Edition) diagnostic criteria, and a previous diagnosis of T2DM. BMI was calculated as the ratio of weight to height squared, and the waist circumference (WC) was measured using a tailor's tape measure (ie, flexible but not stretchable), or similar tape measures, on a patient placed in orthostatism and with the abdomen completely uncovered by clothing.

From the blood tests performed on admission, the following were collected: inflammation indices (erythrocyte sedimentation rate [ESR] and C-reactive protein [CRP]), glycemic balance (basal glucose, insulin, and glycated hemoglobin A_1c_ [HbA_1c_]), lipid profile (total cholesterol, high-density lipoprotein, and triglycerides), hormone profile (TT, luteinizing hormone [LH] and follicle-stimulating hormone [FSH]). All samples for TT and gonadotropin determination were collected between 8 and 10 Am. During admission, if OSAS had never been diagnosed, patients underwent polysomnography, and the AHI values were collected. All polysomnographic monitoring was performed with the same equipment, namely a SOMNOscreen (SOMNOmedics).

On the other hand, if OSAS had already been diagnosed, overnight pulse oximetry was performed under CPAP ventilatory support, and ODI values were collected. Each overnight pulse oximetry record was performed with the PULSOX pulse oximeter (Konica Minolta Inc).

In a subset of 14 newly diagnosed patients with OSAS who started CPAP therapy, TT and BMI were measured after 3 months of successful CPAP therapy (ODI <15) to assess the independent effect of the treatment on the gonadal function.

### Assays

T was evaluated using the Elecsys Testosterone II assay (Calibrator reference: 05200067190) (Roche catalog No. 07027915190, RRID: AB_3101983) manufactured by Roche Diagnostics, which has a lower limit of detection of 0.087 nmol/L and a functional sensitivity of 0.4 nmol/L, with a measurement range of 0.087 to 52.0 nmol/L. The assay method has a cross-reactivity of 2.5% for androstenedione; 0.01% for cortisol, dehydroepiandrosterone, dehydroepiandrosterone sulfate; 0.001% for cortisone, dexamethasone, and progesterone; 0.5% for danazol; 0.16% for estradiol; 0.004% for estrone; 2.4% for etisterone; 0.9% for norgestrel; 2.46% for T propionate; 0.86% for dihydrotestosterone; 18% for 11-B-hydroxytestosterone; 3.22% for 11-ketotestosterone; 6% for 10-norethisterone; and 0.002% for prednisone. This method is standardized by liquid chromatography/mass spectrometry. LH and FSH concentrations were measured by Elecsys LH (Roche catalog No. 07027575190, RRID: AB_2920601) and Elecsys FSH (Roche catalog No. 09745840, RRID: AB_3678555) from Roche Diagnostics. The LH and FSH assays had a minimum detection limit of 0.1 IU/L and a functional sensitivity of 0.2 IU/L, with a measurement range of 0.1 to 200 IU/L. In the FSH assay method there is a cross-reactivity of <0.1% for LH, thyrotropin, human chorionic gonadotropin, human growth hormone, and human placental lactogen. The LH assay method has a cross-reactivity of <0.1% for FSH, thyrotropin, human chorionic gonadotropin, human growth hormone, and human placental lactogen. Elecsys insulin (Roche catalog No. 07027559, RRID: AB_2909455) was used to measure insulin, and CRP levels were assessed by enzyme-linked immunosorbent assay kits (Hytest catalog No. 4C28cc-CRP135cc, RRID: AB_2889099).

### Statistical Analysis

Results are presented as count (%) or mean ± SD depending on the data type and distribution. Groups were compared with nonparametric tests (Mann-Whitney *U* test and Kruskal-Wallis test). A multiple linear regression was also performed to assess the relationship between the progressive reduction of blood values of T and the following independent variables: BMI, severe/decompensated OSAS, previous diagnosis of eating disorder, age, blood values of CRP, and diagnosis of T2DM. All *P* values <.05 were considered statistically significant. To evaluate the effect of CPAP therapy on the hormone profile, T values preventilatory and postventilatory treatment were analyzed with the Wilcoxon signed rank test and a multiple linear regression was finally performed to correlate serum T levels with ODI adjusting for BMI. All data were analyzed using IBM SPSS Statistics 29.0.

## Results

**Figure 1.**
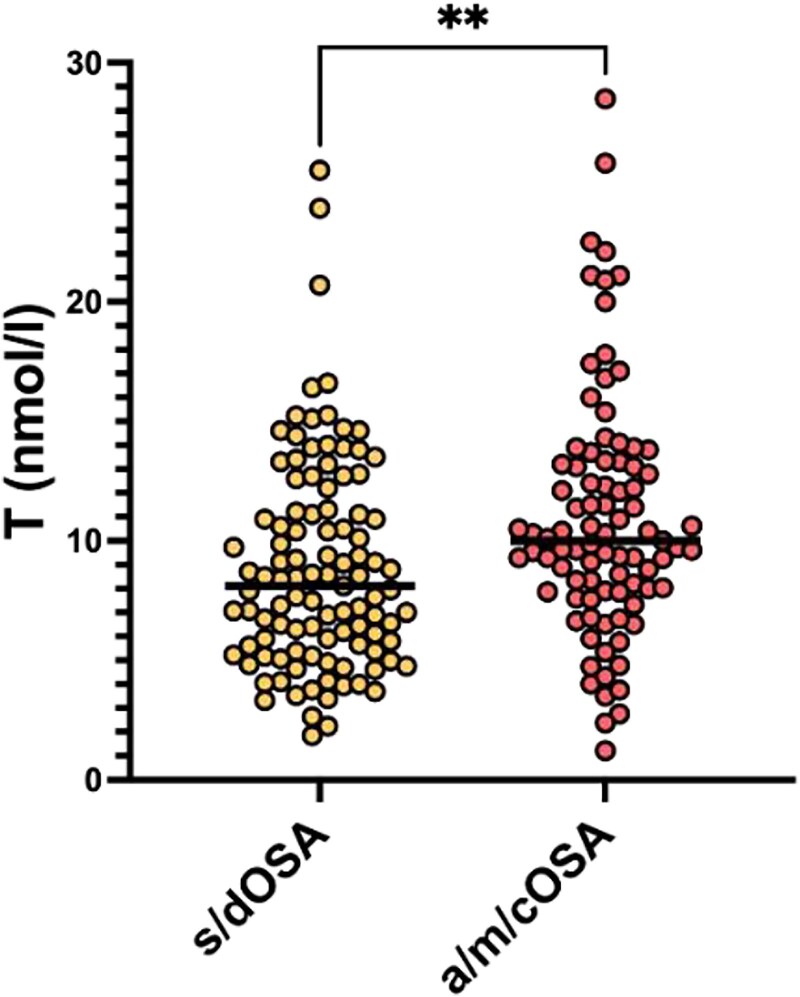
Comparison of testosterone levels between absent or mild-moderate/compensated OSAS and severe/decompensated OSAS. a/m/cOSA, absent/mild-moderate/compensated obstructive sleep apnea syndrome; s/dOSA, severe/decompensated obstructive sleep apnea syndrome; T, testosterone.

The characteristics of the population are shown in Table 1. The population was aged between 18.1 and 87.8 years, with a BMI between 35.39 and 126.4, and a WC between 112 and 215 cm. Among the enrolled patients, 187 patients had either previously been diagnosed or newly diagnosed with OSAS. No patient had central sleep apnea. Eighty-three new diagnoses of OSAS were defined by AHI values at polysomnography performed at admission ≥15 events/hour. Severe/decompensated OSAS (s/dOSAS), defined as AHI (or ODI) above 30 events/hour, was reported in 112 patients undergoing either polysomnography or overnight pulse oximetry during admission. Overall, the study population showed indices of inflammation compatible with low-grade inflammation. A total of 127 patients met the criteria for low T levels (TT ≤ 10.4 nmol/L), according to the diagnostic criterion adopted in previous studies and accepted worldwide for biochemical assessment of hypogonadism in men (16-18)). Among them, 45 individuals (35.4%) showed a marked reduction of gonadal function (TT < 6.0 nmol/L). A total of 76.6% of patients with hypoandrogenemia and concomitant evaluation of gonadotropins had a hypogonadotropic form (LH < 9.4 mU/L). In a subgroup of 51 patients in whom SHBG measurement was available, the mean concentration of this protein in patients with s/dOSAS was comparable to that in the rest of the population (31.6 nmol/L ± 13.21 vs 32.23 nmol/L ± 11.94; *P* = .79). Patients were divided according to AHI or ODI values into 2 groups: absent or mild-moderate/compensated OSAS (AHI/ODI < 30/hour) and severe/decompensated OSAS (s/dOSAS, AHI/ODI ≥ 30/hour). Patients with s/dOSAS were younger, had higher BMI, WC, and CRP levels, but a lower prevalence of T2DM when compared with absent/mild/compensated OSAS patients (see Table 1). Also, TT levels were significantly lower in s/dOSAS (*P* = .002) (Fig. 1). No difference in TT was found when we classified patients simply according to the presence or absence of a diagnosis of OSAS (*P* = .951). We repeated the comparative statistical analysis for T levels after breaking the population down into 3 groups (no OSAS, mild OSAS, and severe OSAS), using the Kruskal-Wallis test. The difference in TT among the groups remained statistically significant (*P* = .003).

**Table 1.** General characteristics of the study population

	Total cohort (204)	Absent or m/cOSAS (92)	s/dOSAS (112)	*P*
Age, y	56.71 ± 13.58	57.13 ± 13.34	55.90 ± 12.36	.017
Anthropometry and clinical history				
BMI	47.45 ± 10.97	45.05 ± 6.32	49.56 ± 11.86	<.001
WC	141.87 ± 15.47	137.86 ± 12.95	145.32 ± 16.60	.001
T2DM	83/204 (40.68%)	39 (43.39%)	44 (39.28%)	.017
BED	45/204 (22.05%)	21 (22.82%)	24 (21.42%)	.92
OSAS	187/204 (91.66%)	—	—	—
s/dOSAS	112/204 (54.90%)	—	—	—
Biochemistry				
CRP, mg/dL	0.87 ± 0.49	0.77 ± 2.47	0.97 ± 2.73	.015
ESR, mm/h	38.20 ± 23.81	40.35 ± 23.23	44.28 ± 25.20	.063
FPG, mg/dL	116.96 ± 38.51	111.72 ± 31.18	120.04 ± 40.51	.216
HbA_1c_, mmol/mol	47.51 ± 17.77	45.64 ± 15.93	48.48 ± 18.51	.245
Insulin, µIU/mL	21.58 ± 12.12	20.9 ± 12.78	22.28 ± 11.78	.442
Total cholesterol, mg/dL	170.29 ± 42.64	159.09 ± 38.34	187.02 ± 42.32	.001
HDL, mg/dL	40.16 ± 16.33	39.72 ± 9.47	40.82 ± 11.35	.382
Triglycerides, mg/dL	166.31 ± 76.01	99.41 ± 75.57	113.92 ± 72.65	.044
Total testosterone, nmol/L	9.77 ± 4.85	9.78 ± 5.12	8.38 ± 4.41	.002
LH, mU/L	7.58 ± 4.76	7.44 ± 3.83	7.66 ± 5.26	.69
FSH, mU/L	7.3 ± 6.01	7.92 ± 6.72	6.2 ± 4.52	.34

Data are expressed as mean SD ± or number (percentage). The Mann-Whitney *U* test was implemented to compare groups.

Abbreviations: BED, binge-eating disorder; BMI, body mass index; CRP, C-reactive protein; ESR, erythrocyte sedimentation rate; FPG, fasting plasma glucose; FSH, follicle-stimulating hormone; HbA_1c_, glycated hemoglobin A_1c_; HDL, high-density lipoprotein; LH, luteinizing hormone; m/cOSAS, mild-moderate/compensated obstructive sleep apnea syndrome; OSAS, obstructive sleep apnea syndrome; s/dOSAS, severe/decompensated obstructive sleep apnea syndrome; T2DM, type 2 diabetes mellitus; WC, waist circumference.

In multiple linear regression analysis, after adjustment for age, BMI, T2DM, BED, and CRP values, s/dOSAS was still significantly associated with TT levels (*P* = .014). Also, BMI, T2DM, and CRP blood concentrations were found to be in significant correlation with blood TT concentrations (*P* = .039; *P* = .006; and *P* = .003, respectively). The results of the aforementioned model are shown in Table 2. These associations were also confirmed when excluding from the analysis the forms of hypergonadotropic hypogonadism (LH ≥ 9.4 mU/L), which accounted for the minority of patients with hypoandrogenemia.

**Table 2.** Factors associated with blood total testosterone concentrations

	TT, total cohort	
	β	*P*
Age	0.009	.906
T2DM	−0.185	.**006**
BED	−0.129	.070
BMI	−0.150	.**039**
CRP	−0.207	.**003**
s/dOSAS	−0.165	.**014**

The statistical analysis was performed using multiple linear regression. Bold values indicate statistical significance (*P* < .05).

Abbreviations: BED, binge-eating disorder; BMI, body mass index; CRP, C-reactive protein; s/dOSAS, severe/decompensated obstructive sleep apnea syndrome; T2DM, type 2 diabetes mellitus; TT, total testosterone.

**Figure 2.**
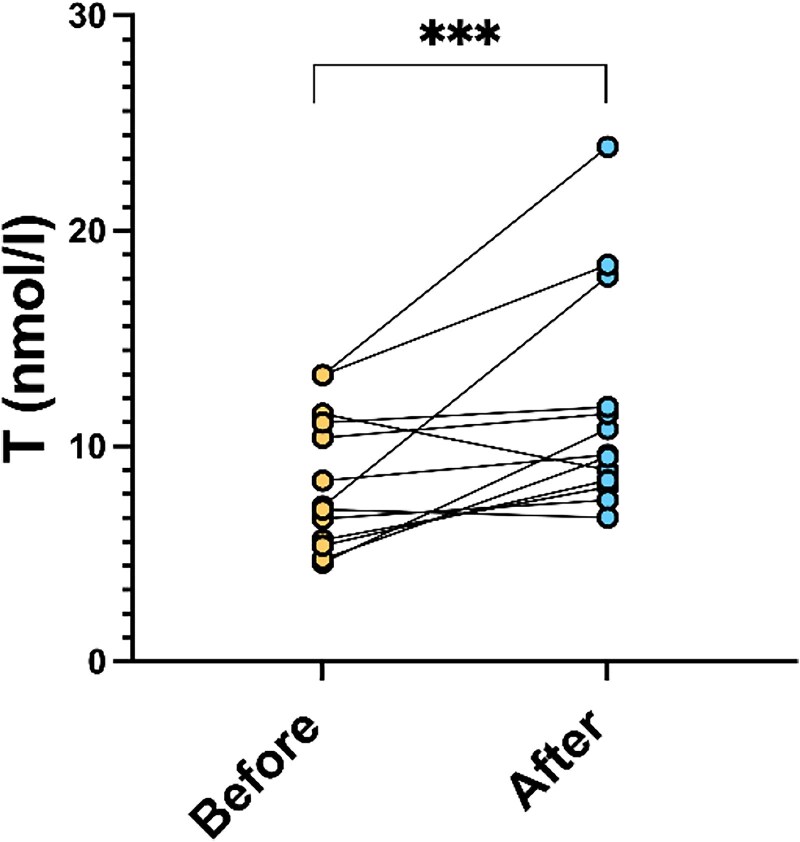
Testosterone values before and after 3 months of continuous positive airway pressure (CPAP) therapy. T, testosterone.

**Figure 3.**
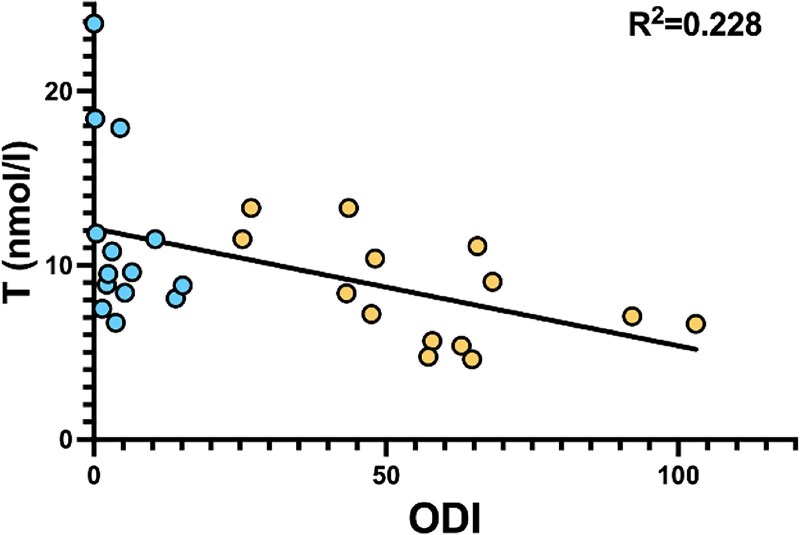
Correlations between total testosterone with oxygen desaturation index in patients undergoing successful continuous positive airway pressure (CPAP) therapy. ODI, Oxygen Desaturation Index; T, testosterone. Yellow spots: before successful CPAP therapy; blue spots: after successful CPAP therapy.

After 3 months of CPAP therapy, T values were found to be significantly improved (Fig. 2; *P* = .009), with an average increase of 3.75 nmol/L (±4.11 nmol/L). Moreover, there was a statistically significant negative correlation between TT and ODI (*P* = .04) levels, independently of BMI, in multiple linear regression (Fig. 3). Every patient had an ODI on CPAP therapy of <15 events/hour, so CPAP therapy was considered successful.

Eight of the 14 patients analyzed longitudinally had baseline gonadotropin assessments. The mean LH and FSH values were 8.02 mU/L (±2.14 mU/L) and 7.32 mU/L (±6.32 mU/L), respectively, and only 1 patient out of 8 had hypergonadotropic hypogonadism (LH = 12.9 mU/L).

## Discussion

To the best of our knowledge, this is the largest study conducted on male patients with severe obesity with the aim of investigating the contribution of OSAS to functional hypogonadism. It appears, in fact, that hypogonadism in patients with obesity is multifactorial. It is well known that BMI and WC correlate with the severity of hypogonadism (19), and alterations in glucose metabolism, which often accompany severe obesity as part of metabolic syndrome (hence the term “*diabesity*”), are also associated with decreased T levels (20). Consistently, T2DM independently correlated with TT in our study, suggesting an additional mechanism in the reduction of T production.

In the dysmetabolic patient, it was initially hypothesized that a suppressive effect on the HPG axis may be exerted by higher levels of circulating estrogens due to increased aromatase activity in adipose tissue. Recent evidence, however, has pointed to dysregulated insulin and leptin signaling, via effects on KNDy (kisspeptin/neurokinin B/dynorphin) neurons in the arcuate nucleus of the hypothalamus (6), and low-grade inflammation, as the origin of HPG axis dysfunction resulting in decreased gonadotropin secretion (21). Indeed, some authors have described that a high-fat diet determined significant microglial activation, expression and immunopositivity of interleukin-6, and reduced immunopositivity for the KISS1 receptor in the hypothalamus of rabbits (22).

Our results are consistent with these data, showing that hypoandrogenemia had a high prevalence in patients with obesity, and T2DM and CRP blood concentrations were independently associated with lower levels of TT in this population. However, as is typical of functional forms, most had slightly reduced T levels, and only a smaller but still significant percentage of individuals had severe hypogonadism (23, 24).

Also, sleep breathing disorders are directly caused by obesity: Increased fat mass, particularly in the neck, and reduced chest wall lung compliance lead to repeated episodes of apnea during sleep. The resulting recurrent hypoxia and associated activation of the sympathetic nervous system and other stress responses can ultimately contribute to higher rates of hypertension, metabolic syndrome, and T2DM, making OSAS an important condition that further complicates organ dysfunction in such patients and requires appropriate management in rehabilitation treatments of obesity (25).

Whether OSAS also contributes to gonadal dysfunction, on the other hand, is still controversial: Several studies have investigated a possible association between this syndrome and male hypogonadism with contradictory results (8-10, 12, 13, 15, 26, 27); however, only a few of these studies have focused on a homogeneous cohort of only patients with severe/complicated obesity and these have often been conducted on limited samples (9, 10, 12). Nevertheless, these 3 studies highlighted the significant association of apnea severity with a decrease in blood T concentration, independently of BMI and, in the study by Tančić-Gajić and colleagues (12), also of metabolic syndrome. Some authors have tried to explain the possible mechanisms underlying this association: Sleep fragmentation in individuals who did not show rapid-eye-movement sleep disrupted the T rhythm with a considerable attenuation of its nocturnal rise (28). In addition, hypoxia may also be responsible for the central suppression of HPG in individuals with OSAS (13) or other lung diseases (29).

Thus, it could be hypothesized that resolution of sleep fragmentation and nocturnal hypoxia by CPAP therapy could lead to an improvement in T levels. However, most studies did not confirm this benefit in patients with OSAS, as evidenced by 2 meta-analyses (15, 30). However, among the included papers, the majority was represented by observational studies with a low number of participants and a short duration of follow-up; moreover, only a few studies reported adequate CPAP use. In our longitudinal study, on the other hand, we included only patients who had good compliance with therapy, finding not only an improvement in T levels after treatment, but also that ODI (measured by overnight pulse oximetry under CPAP therapy) negatively correlated with TT values, even after adjustment for BMI. This finding is particularly relevant since adequate CPAP therapy appears to improve T levels in patients with severe OSAS, with potential benefits on several grounds, including sexual and bone health.

We acknowledge some limitations: A complete assessment of sex hormone profiles was possible in only a subgroup of our population, since SHBG (and calculated FT), gonadotropins, and estradiol measurements were available in only some of the patients, and hypogonadism-related manifestations were not systematically collected. Moreover, the longitudinal study was conducted in only a subset of participants.

Nevertheless, the greatest strength of the study lies in the largest population of patients with obesity ever studied for the correlation between functional hypogonadism and OSAS.

Furthermore, given the monocentric nature of the study, the entire population was studied using uniform procedures and tests. Regarding the lack of calculated FT, we found comparable levels of SHBG among patients with s/dOSAS and absent/mild OSAS: The calculated FT is therefore not expected to show a different trend from that of the TT between the 2 groups. Lastly, to our knowledge, this is the first study to evaluate the effect of CPAP therapy on testosterone values in a cohort of only patients with obesity.

In conclusion, the presence of untreated severe OSAS or OSAS treated with poor disease control contributes to the decrease in T levels in patients with severe obesity. This effect is synergic but independent of other factors impairing HPG function in obesity, such as low-grade inflammation, BMI, and diabetes mellitus. In these patients, proper use of CPAP verified by overnight pulse oximetry has a positive effect on serum T concentrations. In patients with obesity-related functional hypogonadism, a successful treatment of OSAS could help, along with weight loss and management of metabolic alterations, to restore proper gonadal function.

## Data Availability

Original data generated and analyzed during this study are included in this published article or in the data repositories listed in “References.”

## Abbreviations

AHI: Apnea/Hypopnea Index
BED: binge-eating disorder
BMI: body mass index
CPAP: continuous positive airway pressure
CRP: C-reactive protein
ESR: erythrocyte sedimentation rate
FPG: fasting plasma glucose
FSH: follicle-stimulating hormone
FT: free testosterone
HbA_1c_: glycated hemoglobin A_1c_
HPG: hypothalamic-pituitary-gonadal
LH: luteinizing hormone
m/cOSAS: mild-moderate/compensated obstructive sleep apnea syndrome
ODI: Oxygen Desaturation Index
OSAS: obstructive sleep apnea syndrome
s/dOSAS: severe/decompensated obstructive sleep apnea syndrome
SHBG: sex hormone–binding globulin
T2DM: type 2 diabetes mellitus
TT: total testosterone
WC: waist circumference
